# Effect of 16% Carbamide Peroxide and Activated-Charcoal-Based Whitening Toothpaste on Enamel Surface Roughness in Bovine Teeth: An In Vitro Study

**DOI:** 10.3390/biomedicines11010022

**Published:** 2022-12-22

**Authors:** Jorge Zamudio-Santiago, Marysela Ladera-Castañeda, Flor Santander-Rengifo, Carlos López-Gurreonero, Alberto Cornejo-Pinto, Ali Echavarría-Gálvez, Luis Cervantes-Ganoza, César Cayo-Rojas

**Affiliations:** 1 Grupo de Investigación Salud y Bienestar Global, Faculty of Dentistry and Postgraduate School, Universidad Nacional Federico Villarreal, Lima 15001, Peru; 2 Academic Program of Dentistry, Universidad Peruana de Ciencias Aplicadas, Lima 15023, Peru; 3 School of Stomatology, Universidad Científica del Sur, Lima 15067, Peru; 4 School of Stomatology, Universidad Privada San Juan Bautista, Lima 15067, Peru; 5 Faculty of Stomatology, Universidad Inca Garcilaso de la Vega, Lima 15084, Peru

**Keywords:** dental bleaching, toothpaste, carbamide peroxide, activated charcoal, bovine teeth, in vitro study

## Abstract

Background: Activated charcoal is a nanocrystalline form of carbon with a large specific surface area and high porosity in the nanometer range, having consequently the capacity to absorb pigments, chromophores, and stains responsible for tooth color change, while carbamide peroxide is unstable and breaks down immediately upon contact with tissue and saliva, first dissociating into hydrogen peroxide and urea and subsequently into oxygen, water, and carbon dioxide. Therefore, the aim of the present study was to assess the effect of 16% carbamide peroxide and activated-charcoal-based whitening toothpaste on enamel surface roughness in bovine teeth. Materials and Methods: The present experimental in vitro, longitudinal, and prospective study consisted of 60 teeth randomly distributed in six groups: A: artificial saliva, B: conventional toothpaste (Colgate Maximum Protection), C: whitening toothpaste with activated charcoal (Oral-B 3D White Mineral Clear), D: 16% carbamide peroxide (Whiteness Perfect 16%), E: 16% carbamide peroxide plus conventional toothpaste (Whiteness Perfect 16% plus Colgate Maximum Protection), and F: 16% carbamide peroxide plus whitening toothpaste with activated charcoal (Whiteness Perfect 16% plus Oral-B 3D White Mineral Clear). Surface roughness was assessed with a digital roughness meter before and after each treatment. For the statistical analysis, Student’s *t* test for related samples was used, in addition to the ANOVA test for one intergroup factor, considering a significance level of *p* < 0.05. Results: The surface roughness variation of bovine tooth enamel, before and after application of bleaching agent, was higher in groups of whitening toothpaste with activated charcoal (0.200 µm, Confidence Interval (CI): 0.105; 0.296 µm) and 16% carbamide peroxide plus whitening toothpaste with activated charcoal (0.201 µm, (CI): 0.092; 0.309 µm). In addition, bovine teeth treated with conventional toothpaste (*p* = 0.041), whitening toothpaste with activated charcoal (*p* = 0.001), and 16% carbamide peroxide plus whitening toothpaste with activated charcoal (*p* = 0.002) significantly increased their surface roughness values. On the other hand, significant differences were observed when comparing the variation in surface roughness between the application of artificial saliva (control) and the whitening toothpaste with activated charcoal (*p* = 0.031), and the 16% carbamide peroxide plus whitening toothpaste with activated charcoal (*p* = 0.030). Conclusion: The use of whitening toothpaste with activated charcoal and in combination with 16% carbamide peroxide significantly increased enamel surface roughness in bovine teeth.

## 1. Background

Dental beauty currently establishes certain esthetic standards, for which dentistry proposes tooth bleaching as a non-invasive and conservative esthetic procedure in contrast to treatments that involve tooth structure wear. This technique involves the use of different substances that act on the tooth in order to change its shade to lighter colors, increasing its visual whiteness [1,2,3]. As a result, esthetic balance is restored using techniques with minimal loss of tooth structure, achieving significant, fast, and efficient changes [4].

Carbamide peroxide is unstable and breaks down immediately upon contact with tissue and saliva, first dissociating into hydrogen peroxide and urea and subsequently into oxygen, water, and carbon dioxide [4,5,6]. Hydrogen peroxide is a transparent solution capable of penetrating enamel and dentin due to its molecular weight, in addition to its ability to oxidize a wide range of organic and inorganic compounds, producing discoloration and consequent bleaching of the substrate [3,5]. Among its most notable adverse effects, it can produce burns on contact with soft tissues and generate post-treatment sensitivity [4,5]. These side-effects could also damage the tooth surface, producing alterations in the enamel surface roughness. In this regard, it has been reported that a rough surface generates several complications over time such as enamel pigmentation, retention, and accumulation of bacterial plaque on enamel or restorations, which could facilitate the formation of secondary caries, gingival inflammation, and irritation of the tongue, lips, and cheeks [7,8,9,10]. In contrast, smooth surfaces reduce plaque accumulation, recurrent caries, bacterial adhesion, loss of gloss, and long-term discoloration of the natural healthy or restored tooth [7,9].

Recently, whitening toothpastes containing activated charcoal have become popular oral hygiene products, aiming to improve the removal of extrinsic stains and achieve “tooth bleaching”. Toothpastes containing activated charcoal work in a similar way to regular toothpastes [11].

Activated charcoal is a nanocrystalline form of carbon with a large specific surface area and high porosity in the nanometer range, having consequently the capacity to absorb pigments, chromophores, and stains responsible for tooth color change [12]. Because of this ability, charcoal-based toothbrushing can absorb extrinsic stains from the teeth in their pores and change the tooth color [13]. However, there is insufficient scientific evidence for this action and, as a result, it has been assumed that charcoal does not modify tooth color other than by an abrasive action similar to regular toothpastes [14]. For this reason, some authors recommend this type of toothpaste only for color maintenance by delaying the recurrence of surface stains on tooth surfaces after tooth bleaching treatment [12,13]. In addition, it has been proposed that the high absorption capacity of activated charcoal may reduce the availability of fluoride ions in the toothpaste formulation, leading to a limited ability to remineralize tooth tissues and, as a consequence, lower the resistance to caries and tooth decay [15].

Currently on the market, there are a variety of bleaching agents such as hydrogen peroxide, carbamide peroxide, sodium perborate, and activated charcoal that come as outpatient forms in powder and liquid presentations [16,17]. It is very important to know and assess the effects of carbamide peroxide and whitening toothpaste on enamel, allowing the operator to choose an adequate and innocuous treatment option, as, in addition to obtaining the bleaching effect, it is necessary to ensure preservation and integrity of dental hard tissues.

At present, there is little evidence in the scientific literature that has assessed enamel surface roughness following the use of a carbamide peroxide bleaching agent alone or in combination with an activated-carbon-based whitening toothpaste versus a control group. Most studies have evaluated different mechanical properties of either whitening agents or whitening toothpastes, but independently of each other [18,19,20].

Therefore, the present study aimed to assess the effect of 16% carbamide peroxide and activated-charcoal-based whitening toothpaste on enamel surface roughness in bovine teeth. The null hypothesis formulated was that there are no significant differences in the surface roughness of bovine dental enamel when comparing the effect of 16% carbamide peroxide with activated-charcoal-based whitening toothpaste.

## 2. Materials and Methods

### 2.1. Study Design

The present experimental in vitro, longitudinal, and prospective study was conducted at the Universidad Nacional Federico Villarreal (UNFV) and at the Certified High Technology Laboratory (ISO/IEC Standard: 17025), Lima, Peru, from January to March 2022. This study was exempted from review by the ethics committee of the Faculty of Dentistry UNFV. However, its execution was authorized with official letter No.006-2022-COVID-FO-UNFV, as bovine teeth were donated for research purposes by a local certified slaughterhouse authorized by a veterinarian with license CMVP-11107, upon request of the UNFV degree and graduate management office with letter No. 002-2022-OGYGE-FO-UNFV. All methods were performed according to the relevant guidelines and regulations such as the Revised Animals (Scientific Procedures) Act 1986 in the UK and Directive 2010/63/EU in Europe. In addition, the CRIS Guidelines (Checklist for Reporting In Vitro Studies) were considered in this study [21].

### 2.2. Sample Calculation and Selection

**Figure 1:**
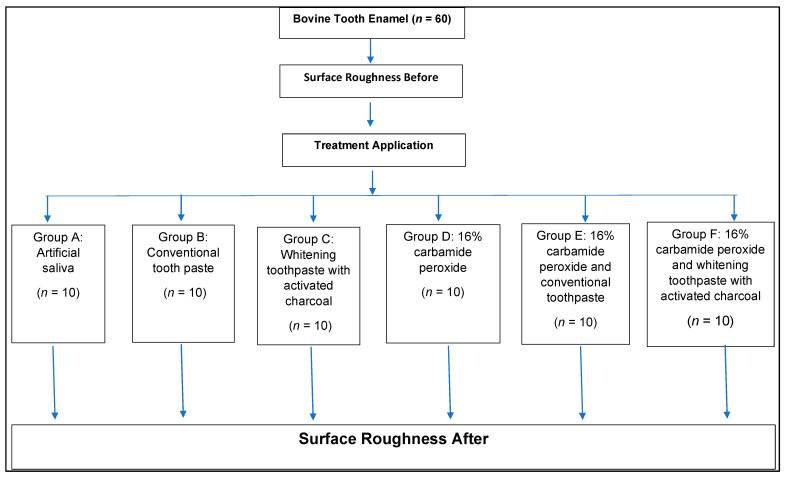
Random distribution of groups, according to sample size.

The total sample size (*n* = 60) was calculated based on data obtained in a previous pilot study with five samples per group, where the analysis of variance formula was applied in the statistical software G*Power version 3.1.9.7 considering a significance level (α) = 0.05 and a statistical power (1 − β) = 0.80, with an effect size of 0.559 and with 6 groups and 2 paired measures. The sampling units were distributed in a simple randomized manner without replacement according to the 6 treatment groups (Figure 1).

A: 4.2 g/L of sodium bicarbonate, 0.5 g/L of sodium chloride, and 0.2 g/L of potassium chloride dissolved in double-distilled water, pH: 7.3 [22,23] (Salival^®^, Laboratorios Unidos S.A., Lima, Peru),

B: Conventional toothpaste (Maxima Proteção Anticáries^®^, Colgate Palmolive Company, São Paulo, SP, Brazil).

C: Whitening toothpaste with activated charcoal (Oral-B 3D White Mineral Clean^®^, Procter & Gamble Company, Greensboro, NC, USA).

D: 16% carbamide peroxide (Whiteness Perfect^®^, FGM Dental Products, Joinville, SC, Brazil).

E: 16% carbamide peroxide plus conventional toothpaste (Whiteness Perfect 16% plus Colgate Maximum Protection).

F: 16% carbamide peroxide plus whitening toothpaste with activated charcoal (Whiteness Perfect 16% plus Oral-B 3D White Mineral Clear).

### 2.3. Sample Characteristics and Preparation

Bovine teeth extracted in the last month prior to the experiment were kept submerged in artificial saliva with replacement every 5 days. The roots were cut with a cooled diamond saw (KG Sorensen, Ind. Com. Ltda.; Barueri, SP, Brazil) in a low-speed turbine (Isomet 1000^®^, Buehler, IL, USA), 2 mm below the amelo-cement junction. Then, cuts were made in the central region of the crown to obtain an enamel block 7 mm long × 4 mm wide × 3 mm thick for each tooth. Then, they were polished with silicon carbide papers No. 400, 600, and 1200 (Carborundum/3M do Brasil Ltda., Sumaré, SP, Brazil) under constant water flow. In addition, an ultrasonic washer cleaning (Codyson^®^, Codyson Electrical Co., Ltd., Shenzhen, China) was performed with distilled water for 10 min. They were stored in a 0.1% thymol solution (Farmacia Universal, Lima, Peru) at 4 °C and were finally randomly distributed in six groups (Figure 1).

### 2.4. Mechanical Brushing Protocol

With the enamel surface facing up, previously fixed on an acrylic block, the corresponding toothpaste was placed with an applicator (Cavibrush^®^, FGM Dental Products, Joenvile, SC, Brazil) on that surface and aided with a mechanical brushing machine (Cycler Equipment, HTL Certificate, Lima, Peru), where 420 brushing cycles (840 passes) were performed with a frequency of 5 Hz and under a load of 200 g or 1.9 N. The samples were brushed with a soft nylon brush (Colgate Twister, Colgate-Palmolive, São Paulo, SP, Brazil). After the brushing cycles, the samples were washed with ordinary water, dried with paper towels, and stored in artificial saliva with replacement every 2 days.

### 2.5. Bleaching Protocol

An applicator was used to place 16% carbamide peroxide on the enamel surface of the samples. Bleaching was performed for 14 days (4 h per day) [14]. An amount of 0.01 g of gel was applied to the enamel surface of samples. The gel was removed from the surface with purified water at the end of each session. The samples were then stored in artificial saliva between 24 h bleaching intervals at 37 °C and renewed each 2 days.

### 2.6. Surface Roughness Test

Once the 60 bovine enamel samples were distributed, surface roughness was measured before the polishing procedure was carried out. After that, the samples were stored for 24 h at 37 °C. The next day, the treatment assigned to each group was started for 14 days. At the end of the treatments, surface roughness was measured again. The surface roughness value was determined as the average of absolute roughness (Ra) in microns (µm) of three measurements taken on each bovine tooth enamel block using a digital roughness meter with a resolution of 0.001 µm (SRT-6200^®^, Huatec, Beijing, China), calibrated at a 0.25 mm cut-off, and 0.2 mm/s speed [7,14].

### 2.7. Statistical Analysis

The data collected were recorded in a Microsoft Excel 2019^®^ file and subsequently imported for statistical analysis by the SPSS program (Statistical Package for the Social Sciences Inc. IBM, NY, USA) version 24.0. For the descriptive analysis, measures of central tendency and dispersion, such as mean and standard deviation, were used. For the inferential analysis, the Shapiro–Wilk test was used to evaluate whether the data had a normal distribution, the Levene homoscedasticity test was used to evaluate the homogeneity of variances, and the Wald–Wolfowitz test was used to analyze the randomness of sample units based on the mean. When verifying that these three statistical assumptions were met, it was decided to use the parametric Student’s *t* test for related samples, and also the one-factor intergroup ANOVA test. In all comparisons, a significance level of *p* < 0.05 was considered.

## 3. Results

The surface roughness variation of bovine tooth enamel before and after the application of carbamide peroxide and whitening toothpaste and controls was greater in the groups of whitening toothpaste with activated charcoal (0.200 µm, 95% CI: 0.105; 0.296 µm) and 16% carbamide peroxide plus whitening toothpaste with activated charcoal (0.201 µm, 95% CI: 0.092; 0.309 µm). In addition, when analyzing the individual variation in the study groups, before and after the application of whitening agent, whitening toothpaste, and controls, it was observed that bovine teeth treated with conventional toothpaste (*p* = 0.041), whitening toothpaste with activated charcoal (*p* = 0.001), and 16% carbamide peroxide plus whitening toothpaste with activated charcoal (*p* = 0.002) significantly increased their surface roughness values. On the other hand, when comparing intergroups variations in the surface roughness of bovine tooth enamel between whitening agent, whitening toothpaste, and controls, significant differences (*p* = 0.007) were observed in at least two of all groups (Table 1).

When multiple comparisons were made of the variations in surface roughness before and after the application of bleaching agents and controls to bovine tooth enamel, according to Tukey’s post hoc test at 95% confidence, significant differences were only observed between the application of artificial saliva (control) and the whitening toothpaste with activated charcoal (*p* = 0.031), and the 16% carbamide peroxide plus whitening toothpaste with activated charcoal (*p* = 0.030).

## 4. Discussion

It is well known that the use of bleaching agents such as hydrogen peroxide or carbamide peroxide for esthetic tooth whitening treatments in the dental office or at home by the patient produces changes in the surface roughness of the enamel. However, it has also been reported that the use of toothpastes with activated-carbon-based components increases the roughness considerably [14,19]. For this reason, the present study aimed to assess the effect of 16% carbamide peroxide and activated-charcoal-based whitening toothpaste on enamel surface roughness in bovine teeth.

The results of the present study showed a significant increase in enamel surface roughness using whitening toothpaste with activated charcoal and also 16% carbamide peroxide (CP) in combination with activated charcoal toothpaste, which is why the null hypothesis was rejected. These findings become relevant when a significant increase in surface roughness is observed when toothpastes with abrasive components such as activated carbon are used alone or as a complement after home use tooth whitening. This last reference is important as the dental professional as part of the hygienization in the preventive phase of the treatment recommends the patient to complement the brushing technique with the use of toothpastes, but whose components do not increase the surface roughness of the tooth such as hydrated-silica-based toothpastes. In this way, the accumulation of bacterial plaque is not facilitated, but rather the remineralization and integrity of the dental substrate [24,25].

For selection of a whitening agent, two important aspects should be taken into account: the efficacy of bleaching and the risk of side-effects such as enamel surface roughness, tooth sensitivity, and gingival irritation [26]. The presence of significant surface roughness has not been demonstrated in the thinning process with 10% CP, nor even with 35% hydrogen peroxide [27,28,29]. However, other studies have shown morphological changes in the enamel surface after 10% CP bleaching, with increased surface roughness, decreased surface microhardness with adverse changes in the elastic modulus of enamel, random fragmentation of the enamel organic protein matrix, deep crevices, and increased surface corrosivity [30,31,32]. In addition, it is known that teeth with higher surface roughness may be more susceptible to bleaching depending on the concentration and time of application [30].

Although there is a general concept that at-home tooth whitening is more effective and produces less tooth sensitivity than tooth whitening with the dental professional, de Geus et al. [33] could not confirm this in their study, due to the high variability of protocols in both whitening techniques. Differences in the results of the two studies can be attributed to the different methods employed with respect to exposure time, storage medium, pH of the solution, and composition of the whitening agents [14]. Acidic bleaching agents can decrease the microhardness of enamel, resulting in an altered surface morphology [27,30]. However, the remineralizing potential of saliva can counteract the effects that whitening products have on enamel and dentin [34].

The at-home dental whitening technique offers advantages such as easy application, reduced chair time, low cost, high success rate, and safety of the materials used [35,36]. CP is an at-home whitening available with concentrations ranging from 10% to 20%. Regardless of their concentration, these bleaching gels are recommended for periods of 2 to 8 h per day during nighttime [35]. Geus et al. [26], Matis et al. [37], and Leonard et al. [38] in their studies reported that the efficacy of home bleaching with 10% CP produced similar whitening effects to CP agents at higher concentrations, in addition to a lower risk of side-effects and intensity of tooth sensitivity.

The interest of many patients in low-cost, easy-to-apply, and professionally unsupervised tooth bleaching alternatives is creating interest and attention for the use of whitening toothpastes containing organic ingredients such as activated charcoal [33,39,40]. These products can be found in powder form to be used with direct application to the toothbrush, to be mixed with conventional toothpastes and/or whitening toothpastes [14], or in toothpastes with activated charcoal in their composition [19]. Therapeutic properties such as low tooth abrasiveness, remineralization, detoxification action, and antifungal, antibacterial, or antiseptic action were some of the benefits of activated charcoal toothpastes that allowed them to receive the endorsement of the dental professional for commercial use [11,18,41]. However, the benefits of products containing activated charcoal for tooth brushing have also been questioned due to the possible risks of tooth surface wear [39,40,42,43].

The present study showed that the surface roughness of tooth enamel was greater in whitening toothpaste groups with activated charcoal (Oral-B 3D White Mineral Clear) and in the group subjected to 16% CP and whitening toothpaste with activated charcoal (Oral-B 3D White Mineral Clear + Whiteness Perfect 16%), coinciding with the studies carried out by Franco et al. [18], Palandi et al. [14], De Andrade et al. [39], and Pertiwi et al. [44], who reported that activated charcoal in toothpastes increased the surface roughness of enamel, favoring the installation of bacterial plaque, in addition to absorbing and retaining chromophore pigments, causing changes in tooth color. In addition, most of the toothpastes containing activated charcoal may contain an average of 8% fluoride in their composition [41]. However, the presence of activated charcoal inactivates or minimizes the action of fluoride, producing dental wear such as pronounced porosity and visible depressions in the enamel [14,43].

In the study by Franco et al. [18], there was no significant difference in enamel surface roughness for the three groups assessed with conventional toothpaste, powder-based charcoal, and 10% CP, in contrast to the present study in which the difference was significant. This could be due to the fact that the charcoal used by Franco et al. was in powder form and not in toothpaste, in addition to using 36 brushing cycles per minute in their methodology, with an intensity of 0.6 Hz and a charge of 4.5 N [18]. On the other hand, the study by Palandi et al. [14] coincided with the present study, finding high values of enamel roughness in groups subjected to toothpastes containing activated charcoal and groups that used whitening agents in combination with other whitening toothpastes, arguing that this bleaching effect of charcoal on the surface is based on the abrasion caused by brushing movements, and the CP mechanism of action may be responsible for the alterations in roughness [45]. In addition, Palandi et al. [14] used a methodology very similar to the present study in terms of brushing cycles: 412 for 14 days with a frequency of 5 Hz under a load of 200 g, and a CP concentration of 16% (FGM Dental Group, Joinville, Santa Catarina, BR). The combination of CP with brushing based on bleaching agents can compromise the enamel, so conventional toothpastes in combination with CP could be the best option for bleaching effects and not considerable enamel damage. For Singh et al. [42], large alterations in the enamel roughness were represented microscopically as irregular depressions, attributing this to the activated charcoal composition, as it contains kaolin clay, which, in combination with brushing, could generate high abrasiveness.

Conventional toothpastes used in the present study had less abrasive calcium carbonate particles in comparison to whitening toothpastes and those containing activated charcoal, as the latter have additional components such as hydrated silica and titanium dioxide or kaolin. Thus, they produce moderate to severe abrasiveness, which would explain the significant change effects of using whitening toothpastes with activated charcoal or combined with CP-based whitening agents [24].

Vural et al. [25], in their in vitro study on human teeth, found that enamel surface roughness increased with the use of activated-charcoal-based whitening toothpastes, except for Black is White paste. In a review article on whitening toothpastes containing activated charcoal, it was reported that 28% of these toothpastes have low abrasiveness, perhaps answering why Black is White toothpaste showed little change in enamel roughness by having a “low abrasive” ingredient in comparison to other toothpastes with activated charcoal such as Oral-B 3D White Mineral Clean, used in the present study. Melo et al. [24] reported that there were no significant differences in the enamel roughness produced by hydrated-silica-based bleaching toothpastes. These findings can be explained by the methodological heterogeneity applied due to the absence of activated charcoal in all the toothpastes used [19].

The reason why only 420 brushing cycles were applied in the present study design [14,18,20,25] was because some studies reported that longer brushing cycles decrease the enamel surface volume [46]. Silva et al. [46] and Vieira et al. [47], in their studies with bovine teeth, reported that the formation of grooves and roughness on the enamel surface increased significantly and progressively during eight weeks of assessment due to a cumulative effect of tooth brushing and also due to the characteristics of the abrasive particles included in the toothpaste formulation, i.e., hardness, size, shape, and distribution of these particles [13,43,48,49,50,51].

The importance of present study lies in the fact that it provides information on the effects on enamel of whitening agents in combination with whitening toothpastes. In addition, there is a need for further studies that will allow dentists to make an adequate and safe treatment option and achieve not only a long-term esthetic bleaching effect, but also ensure the preservation and integrity of the dental substrates. In present study, the rigorous methodology employed based on scientific antecedents concerning sample size, use of bovine teeth, strict protocol for sample preparation, products used according to antecedents, number of cycles, intensity and load for each brushing, evaluation instrument used, roughness evaluation measurements before and after application of bleaching agent, among others, allowed the reduction in biases and strengthening of the design. However, it should be recognized within the limitations of the present study that the data obtained should be taken with caution because, being an in vitro study, it is not possible to extrapolate it to the clinical field. Nevertheless, the present study lays the foundation for future randomized clinical trials with well-designed protocols to assess the real effects of activated charcoal in combination with carbamide-peroxide-based whitening agents at different concentrations on the adamantine surface. It is also recommended to assess and compare the longevity and color stability, enamel surface morphology, or other mechanical properties when applying the bleaching agents used in this study.

## 5. Conclusions

In summary, considering the limitations of the present in vitro study, whitening toothpaste with activated charcoal and in combination with 16% carbamide peroxide significantly increased enamel surface roughness in bovine teeth, while 16% carbamide peroxide did not cause significant changes in enamel surface roughness. Finally, the use of a bleaching agent before brushing with toothpaste could be a determining factor in the modifications revealed by the roughness parameter.

## Figures and Tables

**Table 1:** Comparison of intragroup and intergroups variation in surface roughness (µm) when applying whitening agent, whitening toothpaste, and controls to bovine tooth enamel.

Bleaching Agent and Controls	*n*	Ⴟi	Ⴟf	(Ⴟ_f_ − Ⴟ_i_)	SD	SE	95% CI		*t*	* *p*	F	** *p*
							LL	UL				
Artificial saliva (control)	10	0.429	0.449	0.020	0.031	0.010	−0.002	0.043	2.084	0.067	3.641	0.007 **
Conventional toothpaste (control)	10	0.355	0.482	0.127	0.169	0.053	0.006	0.248	2.383	0.041 *		
Whitening toothpaste with activated charcoal	10	0.401	0.601	0.200	0.134	0.042	0.105	0.296	4.734	0.001 *		
16% Carbamide Peroxide	10	0.629	0.677	0.048	0.108	0.034	−0.030	0.125	1.392	0.197		
16% Carbamide peroxide plus conventional toothpaste	10	0.444	0.519	0.075	0.127	0.040	−0.016	0.166	1.864	0.095		
16% Carbamide peroxide plus whitening toothpaste with activated charcoal	10	0.599	0.800	0.201	0.151	0.048	0.092	0.309	4.191	0.002 *		

*n*: sample size; (Ⴟf): After; (Ⴟi): Before; (Ⴟf − Ⴟi): Mean difference of surface roughness in µm; SD: Standard Deviation; SE: Standard Error; 95% CI: Confidence interval of the difference at 95%; *t*: Student’s *t*-test for related samples (* *p* < 0.05, significant differences); F: ANOVA test for one intergroup factor (** *p* < 0.05, significant differences in at least two of all groups).

## Data Availability

The data presented in this study are available on request from the corresponding author.
